# Epidemiological analysis of second primary malignant neoplasms in cancer survivors aged 85 years and older: a SEER data analysis (1975–2016)

**DOI:** 10.1038/s41598-022-15746-x

**Published:** 2022-07-08

**Authors:** Xianlan Zhao, Li Zhang, Lanjun Bai, Yangyang Zhao, Qiao Yang

**Affiliations:** 1 People’s Hospital of Honghuagang District, Zunyi, China; 2 Department of Clinical Laboratory, The 941st Hospital of the PLA Joint Logistic Support Force, Xining, China; 3 Department of Urology, The 941st Hospital of the PLA Joint Logistic Support Force, Xining, China; 4 Department of Neurosurgery, Southwest Hospital, Army Medical University, Chongqing, China; 5 Department of Ultrasound, The 941st Hospital of the PLA Joint Logistic Support Force, 67 Bayi East Road, Chengdong District, Xining, 810007 China

**Keywords:** Cancer, Cancer epidemiology

## Abstract

Cancer burden in patients aged 85 years and older has rapidly increased accompanying the decrease in mortality, which is raising the concern of developing second primary malignant neoplasms (SPM). This study aimed to investigate the epidemiology of the SPM in this population in the US by using the surveillance, epidemiology, and end results database (1975–2016). The cumulative incidence of developing a SPM was calculated by the Fine and Gray model. Standardized incidence ratios (SIR) were calculated via Poisson regression. The relative post-SPM survival rate was calculated by the Kaplan–Meier method. Male patients with skin melanoma, kidney and renal pelvis and urinary bladder cancers had high cumulative incidences (15.32%, 13.55%, and 12.26%, respectively) and increased SIRs (1.47, 1.44, and 1.16, respectively) for developing SPMs. Female patients with skin melanoma and urinary bladder cancers had high cumulative incidences (10.18% and 7.87%, respectively) and increased SIRs (1.34 and 1.18, respectively). In general, the incidence of SPM cases increased over time. The median latency ranged from 17 to 37 months. A less than 50% of patients had 1-year post-SPM survival. In conclusion, some of these patients had an increased risk of the SPM, with poor survival.

## Introduction

The burden of cancer in patients aged 85 years and older, also named the oldest old, has been rapidly increasing, but the mortality rate of these patients has decreased^1,2^. In the United States, the number of cancer survivors aged 85 years and older has been estimated to increase from 1.9 million in 2019 to 4.7 million in 2040^1,3^. With the increased incidence and decreased mortality in this population, a second primary malignant neoplasm (SPM) may frequently occur as a late effect of the first primary malignant neoplasm (FPM), exerting a substantial adverse impact on survival^4^. Hence, considerable clinical importance is to be attributed to the assessment of the risk of the SPMs in cancer survivors aged 85 years and older.

Our previous work^5^ provided a general overview of the comprehensive epidemiology profile of SPMs in this population, but specific cancer types were not included in this research. To our best knowledge, no study focuses on the SPM occurrence in the oldest old with specific cancer types. Hence, this study aimed to investigate the epidemiology of the SPMs in 10 leading cancer types occurring in males and females, which were selected from the previous study^1^, using the Surveillance, Epidemiology, and End Results (SEER) database in the US. In addition, the overall risk of SPMs in different FPM and overall post-SPM survival were explored.

## Results

A total number of 52,336 male and 67,754 female patients with cancer and aged 85 years and older were identified from the SEER database between 1975 and 2016, of which 4240 male and 4052 female patients developed a SPM. A comparison was performed between the baseline clinical features of an only one primary malignancy (OPM) group and a SPM group. The results obtained are summarized and presented in Supplementary Tables 1 and 2. Generally, the SPM group was of a low tumor grade, and early SEER stages. In addition, in the SPM group, a higher proportion of patients died of non-cancer-related causes, but a lower proportion died of cancer, as compared to the OPM group.

Among the male patients with the selected FPMs, skin melanoma (15-year, 15.32%) had the highest cumulative incidence of the SPMs, followed by the cancers in the kidney and renal pelvis (15-year, 13.55%) and the urinary bladder cancers (15-year, 12.26%). The top 3 FPMs with highest cumulative incidence in female patients were skin melanoma (20-year, 10.18%), urinary bladder cancer (20-year, 7.87%), and breast cancer (20-year, 7.35%). The data of the cumulative incidence of each FPM are presented in Table 1. The numbers of the observed SPM cases for each FPM during different periods are displayed in Supplementary Fig. 1, where a general increasing trend over time can be observed. For male patients, the increased trends of observed SPM cases were more notably in patients with prostate cancer, urinary bladder cancer, colon and rectum cancer, and melanoma of the skin. For female patients, it was more notably in patients with breast cancer, and colon and rectum cancer.

**Table 1:** Cumulative incidence of second primary malignant neoplasms for selected first primary malignant neoplasms, ages 85 years and older, SEER, 1975–2016.

First primary malignant neoplasms	1-year (%)	3-year (%)	5-year (%)	10-year (%)	15-year (%)	20-year (%)
**Male**						
Lung and bronchus	1.12	2.85	3.66	4.36	4.36	NA
Prostate	1.07	4.19	6.03	7.48	7.70	7.71
Urinary bladder	2.42	6.96	9.87	12.11	12.26	NA
Colon and rectum	1.70	5.14	7.44	9.09	9.39	9.39
Melanoma of the skin	2.63	9.11	12.36	15.15	15.32	NA
Non-Hodgkin lymphoma	1.33	4.83	6.70	8.58	8.76	NA
Leukemia	1.86	5.24	6.84	8.11	8.32	NA
Pancreas	0.25	1.87	2.17	3.17	NA	NA
Kidney and renal pelvis	3.65	9.69	12.27	13.55	13.55	NA
Stomach	0.73	2.75	3.70	4.53	NA	NA
**Female**						
Breast	0.92	3.47	5.18	6.95	7.29	7.35
Colon and rectum	0.88	3.54	5.01	6.27	6.52	6.55
Lung and bronchus	0.73	2.02	2.77	3.24	3.24	NA
Pancreas	0.12	0.50	0.50	0.70	0.70	NA
Non-Hodgkin lymphoma	0.86	3.47	4.62	5.76	6.09	NA
Urinary bladder	1.25	4.38	5.97	7.67	7.87	7.87
Leukemia	1.05	3.83	5.57	6.31	6.31	NA
Melanoma of the skin	1.79	5.23	7.24	9.77	10.18	10.18
Ovary	0.55	1.61	1.95	3.01	3.13	3.13
Uterine corpus	0.60	2.94	4.45	6.37	6.50	6.50

SEER, surveillance, epidemiology, and end results; NA, not available.

**Figure 1:**
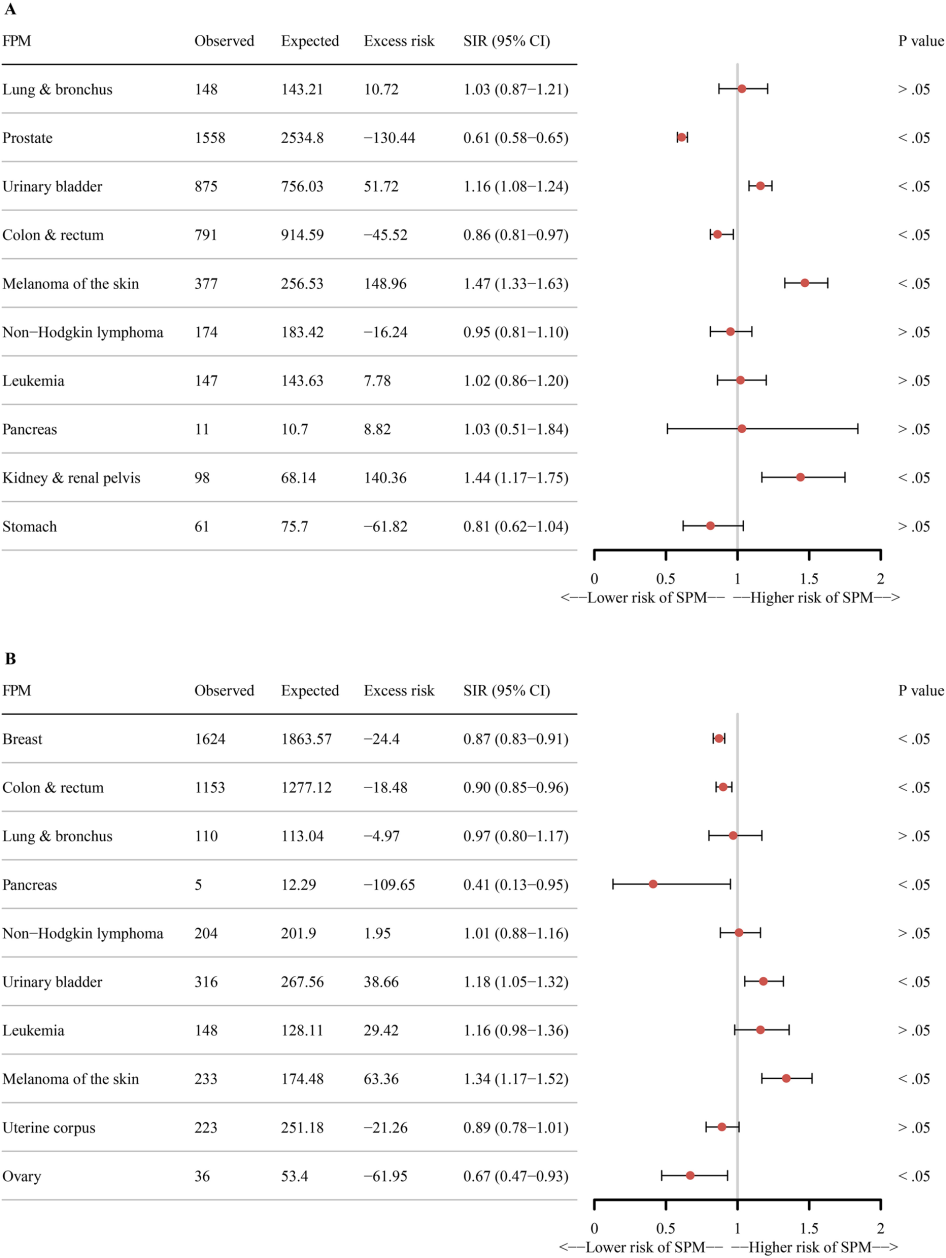
SIRs and 95% CIs for SPM in cancer survivors aged 85 years and older. (**A**) Male patients and (**B**) female patients. The ten leading primary cancer types in males and females aged 85 years and older were selected. The excess risk is per 10,000 persons. SIR > 1.00 indicates a higher risk. SIR, standardized incidence ratio; CI, confidence interval; SPM, second primary malignant neoplasms.

Compared to the US general population for the analysis of the risk of SPMs, the male patients aged 85 years and older with skin melanoma (standardized incidence ratio [SIR] 1.47, 95% confidence interval [CI] 1.33–1.63, *P* < 0.05), kidney and renal pelvis (SIR 1.44, 95% CI 1.17–1.75, *P* < 0.05), and urinary bladder cancers (SIR 1.16, 95% CI 1.08–1.24, *P* < 0.05) were at an increased risk of SPMs. Patients with the prostate (SIR 0.61, 95% CI 0.58–0.65, *P* < 0.05) and colon and rectum (SIR 0.86, 95% CI 0.81–0.97, *P* < 0.05) cancers were at a lower risk of SPMs. Female patients aged 85 years and older, those with skin melanoma (SIR 1.34, 95% CI 1.17–1.52, *P* < 0.05) and urinary bladder cancers (SIR 1.18, 95% CI 1.05–1.32, *P* < 0.05) were at a higher risk of SPMs. Patients with the ovary (SIR 0.67, 95% CI 0.47–0.93, *P* < 0.05), breast (SIR 0.87, 95% CI 0.83–0.91, *P* < 0.05), and colon and rectum (SIR 0.90, 95% CI 0.85–0.96, *P* < 0.05) cancers were at a lower risk of SPMs (Fig. 1).

Cancer survivors with different FPMs had varied SPMs. The three major SPM sites of each FPM are listed in Supplementary Table 3. The most common SPM sites in male patients were the prostate, lungs and bronchus, colon and rectum, and urinary bladder. In female patients, the most common SPM sites were the breast, colon and rectum, and lung and bronchus.

In the analysis of the latency difference (Supplementary Figs. 2, 3), the male prostate cancer (32.0 months) and the female uterine corpus cancer (37.0 months) patients had the longest mean latency to develop a SPM. Conversely, the male pancreas cancer (17.0 months) and the female pancreas cancer (20.0 months) patients had the shortest mean latency. However, the post-SPM survival in this population was poor (Supplementary Table 4). Notably, less than 50% of the patients had 1-year post-SPM survival. The 3-year survival rate ranged from 9 to 28%, and the 5-year survival rate ranged from 6 to 20%.

## Discussion

To the best of our knowledge, this is the first population-based study to present the epidemiological findings of the SPMs in patients with cancer in the US, specifying 10 selected cancer types and an age of 85 years and older.

We established an increased number of SPM cases over time in the male patients with prostate, urinary bladder, and colon and rectum cancers, and the female patients with breast and colon and rectum cancers. The high incidence and relatively higher 5-year survival rates in these cancers might have contributed to obtaining these results^1^. However, the SIRs of the SPM in these cancers were significantly lower, except for urinary bladder cancer in males, and the probable reason might be that the incidences of these cancers decreased rapidly from 1975 to 2015^1^ and were close to the death rates in the last decade. In addition, a previous study explored the SPM incidence among cancer survivors aged ≥ 18 years old in the US. The results turned out that the most common FPM sites were prostate cancer, female breast cancer, colorectal cancer and bladder cancer^6^. These findings suggested that these FPM sites are prone to SPM occurrence, irrespective of age. We also found a significant increase of observed SPM cases of male melanoma patients. The probable reason could be the rapid increase in incidence of male melanoma patients in the oldest old (7.4% increase annually for year 1995–2002, and 4.3% increase annually for year 2002–2015), but relatively stable and low mortality in these patients^1^. While for patients with other FPMs, the observed cases remained relatively stable over years. The most probable reason could be the relatively higher proportion of advanced stages. These patients could be less effectively treated, such as surgery, or untreated, which led to worse survivals^1^. As a result, these FPMs could not have enough time to develop a SPM.

In contrast to the observed SPM cases, we found different trends in SIRs. In this study, most FPMs had decreased or nonsignificant SIRs for SPM occurrence compared to the oldest old US general population. This was consistent with previous studies which demonstrated that the SIRs for SPM occurrence in older cancer survivors were comparable with general older population^7–9^. While for male patients with urinary bladder and kidney cancers, and female patients with urinary bladder cancers, we observed relatively higher SIRs. The probable reason could be that these cancers were smoking-related cancers, which were demonstrated to be at a high risk of SPM occurrence^7^. Though lung cancer is also a smoking-related cancer, these patients had relative worse survival, which means most of these patients would die before developing a SPM. In addition, we observed a risk of SPMs that was 47% higher in male and 34% higher in female skin melanoma cancer survivors in an oldest-old population. Both male and female skin melanoma patients had the highest cumulative incidences of SPMs. The rapidly increased incidence, accompanied by stable low skin melanoma death rates in the oldest-old patients with cancer, might be the possible explanation for this phenomenon^1^. Reportedly, the incidence rate of skin melanoma has been continually increasing through age 95 years and older^10,11^.

The post-SPM survival rate in the oldest-old cancer survivors was extremely poor, with no more than 50% of the patients surviving more than one year. The 5-year survival rate was only from 6 to 20%. Hence, a cancer screening should be performed in this population within a median latency of 17–37 months from the diagnosis of the FPMs. By now, how to effectively treat SPMs are still unclear. No literature focuses on the management of metachronous multiple primaries. Individual therapy considering both the FPM and SPM are recommend, preferably by a multidisciplinary team^5,12^. Specifically, the histology type of each malignancy, the stage of the disease, the patient’s overall health status, whether the SPM could be treated with curative intent, prior treatment regime for FPM, possible complications, carcinogenic factors that can be managed should be taken into consideration^12,13^. In addition, more attention should be paid to the treatment of comorbidities since the likelihood of death from non-cancer-related causes could be higher in the older patients with cancer^14,15^.

## Limitations

The limitations of this study are to be acknowledged. First, the impact of treatment modality on SIR, which was an important influencing factor, was not well addressed. On the one hand, no information was available on surgery treatment before year 1998 in the SEER database. On the other hand, the data concerning radiation and chemotherapy were also incomplete. For patients with unknown radiation or chemotherapy treatment status, it was unknown whether it was received or not by the patient or whether it was only missed in the registry. The lack of adjustment by treatment modality might have caused potential bias. Next, we did not perform SIR risk factor analysis. Relevant explanation and presentation of the results was difficult to provide due to the potential variations in the risk factors in different FPMs. Therefore, this issue should be addressed in future research, which is to be focused only on one FPM site. Then, the above findings are mostly based on Caucasians (88%) in the US, which might not be possible to generalize to other populations or countries. After that, the dataset of this study limited to 1975–2016. Up-to-date findings with most recent data should be performed in future. Last, in this study, the statistical analyses of SIRs were calculated by SEER*Stat software, which only provide the information about whether the statistics were significant or not (i.e. *P* < 0.05 or *P* > 0.05). The calculation of precise *P*-values was not possible because original data was not available.

## Conclusion

This report provides a brief epidemiological profile of the SPMs in the oldest-old patients with a selection of the 10 most common cancer types. Our finding suggest a higher SIR of the SPMs in skin melanoma, urinary bladder and kidney, and renal pelvis cancer patients. The post-SPM survival in the oldest-old cancer survivors was dismal.

## Methods

### Patients

All patients aged 85 years and older at FPM diagnosis, and diagnosed with a known FPM or SPM from 1975 to 2016 were identified from the SEER 9 registries, accounting for approximately 9% of the US population^16^. Based on the results of a previous study^1^, only the leading 10 FPMs in the patients aged 85 years and older were included in this epidemiological analysis. The inclusion of patients with FPM was limited to microscopically confirmed cases. The patients with only autopsy and death certificate were not included in this study. SPM was defined as the subsequent primary malignant neoplasm among cancer survivors. For SPM with the same organ or type as the FPM, the multiple primaries documented in the SEER database were employed to distinguish the SPM from recurrence^17^. Generally, the related factors for distinguish the SPM from recurrence comprised of histology, biology, clinical presentation, etc. In addition, at least a 6-month latency between the FPM and SPM was required because additional cancers occurred within six months were considered to be synchronous or multiple primary tumors according to previous studies^13^. In this study, SEER stage includes four integrated stages, i.e. local stage, regional stage, distant stage and unknown stage. Local stage was defined as an invasive neoplasm confined entirely to the organ of origin. Regional stage was defined as a neoplasm that has extended beyond the limits of the organ of origin directly into surrounding organs or tissues, or into regional lymph nodes by way of the lymphatic system, or by a combination of extension and regional lymph nodes. Distant stage was defined as a neoplasm that has spread to parts of the body remote from the primary tumor either by direct extension or by discontinuous metastasis to distant organs, issues, or via the lymphatic system to distant lymph nodes. For prostate cancer cases, those with local stage or regional stage were integrated into one group, i.e. local/regional group, according to the SEER database^18^.

### Study design and statistical analysis

Baseline clinical features differences, including age at FPM diagnosis, race, FPM site, tumor grade, SEER stage, and cause of death, between patients with OPM and SPM were compared. Age was described as median and interquartile range (IQR) and compared by the Mann–Whitney U test. Other categorical variables were represented as frequencies and compared by Pearson’s *Χ*^2^ test.

The cumulative incidence rate of SPM development was calculated by the Fine and Gray model, considering the patient’s death as a competing event. The SIR, which was defined as the ratio of the observed incidence of SPMs in cancer survivors to the incidence of relative cancers in the US general population, with a 95% CI, was calculated by Poisson regression. The relative post-SPMs survival, which was defined as the follow-up time from the diagnosis of SPMs to the death due to any reason, was calculated by the Kaplan–Meier method. The patients alive at the time of the last follow-up examination (31st December 2016) were regarded as the censored cases. The patients with the unknown survival time (n = 249) were excluded from the survival analysis. In addition, the latency distribution and the number of observed SPM cases during each period were calculated. Two-sided *P* value of < 0.05 was considered to indicate the statistically significant differences. Statistical analyses of SIR, post-SPM survival and leading sites of SPM were calculated by SEER*Stat (version 8.3.9). Other statistical analyses were performed by R software (version 4.0.3).

### Ethics approval

Ethical approval for this study was not required as it is a retrospective analysis of a public dataset. This study was followed the principles outlined in the Declaration of Helsinki for all human investigations.

## Supplementary Information

Supplementary Information 1.

Supplementary Information 2.

Supplementary Information 3.

Supplementary Information 4.

Supplementary Information 5.

Supplementary Information 6.

Supplementary Information 7.

## Data Availability

The datasets for this study can be obtained from the corresponding author upon any reasonable request.
