# Supplementary figures and images for: Epidemiological analysis of second primary malignant neoplasms in cancer survivors aged 85 years and older: a SEER data analysis (1975–2016)
Source: Sci Rep. 2022 Jul 8;12:11688. doi: 10.1038/s41598-022-15746-x (PMC9270446; doi:10.1038/s41598-022-15746-x)

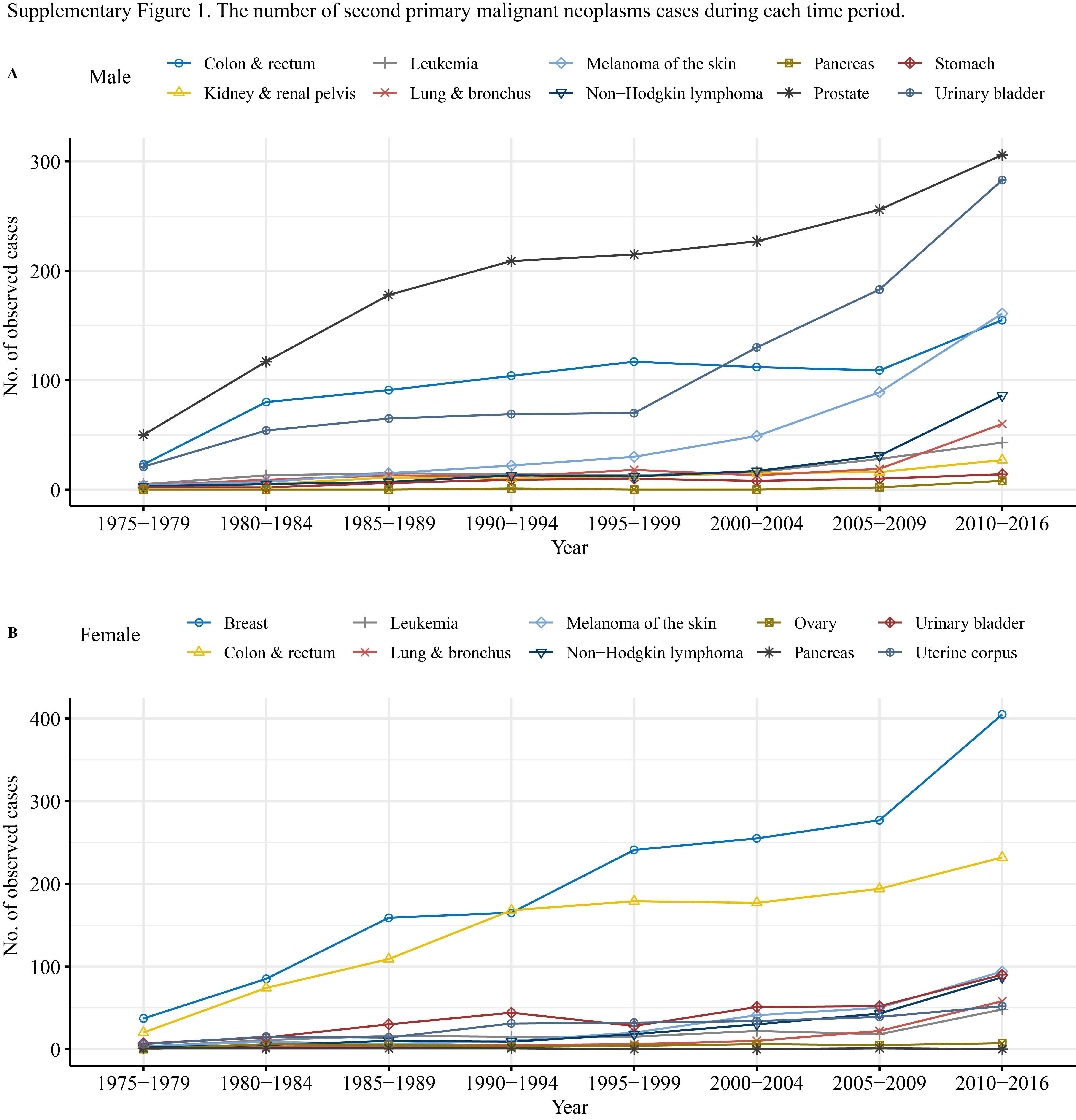

Supplement: Supplementary file 1 — Supplementary Information 1. [file 41598_2022_15746_MOESM1_ESM.jpg]

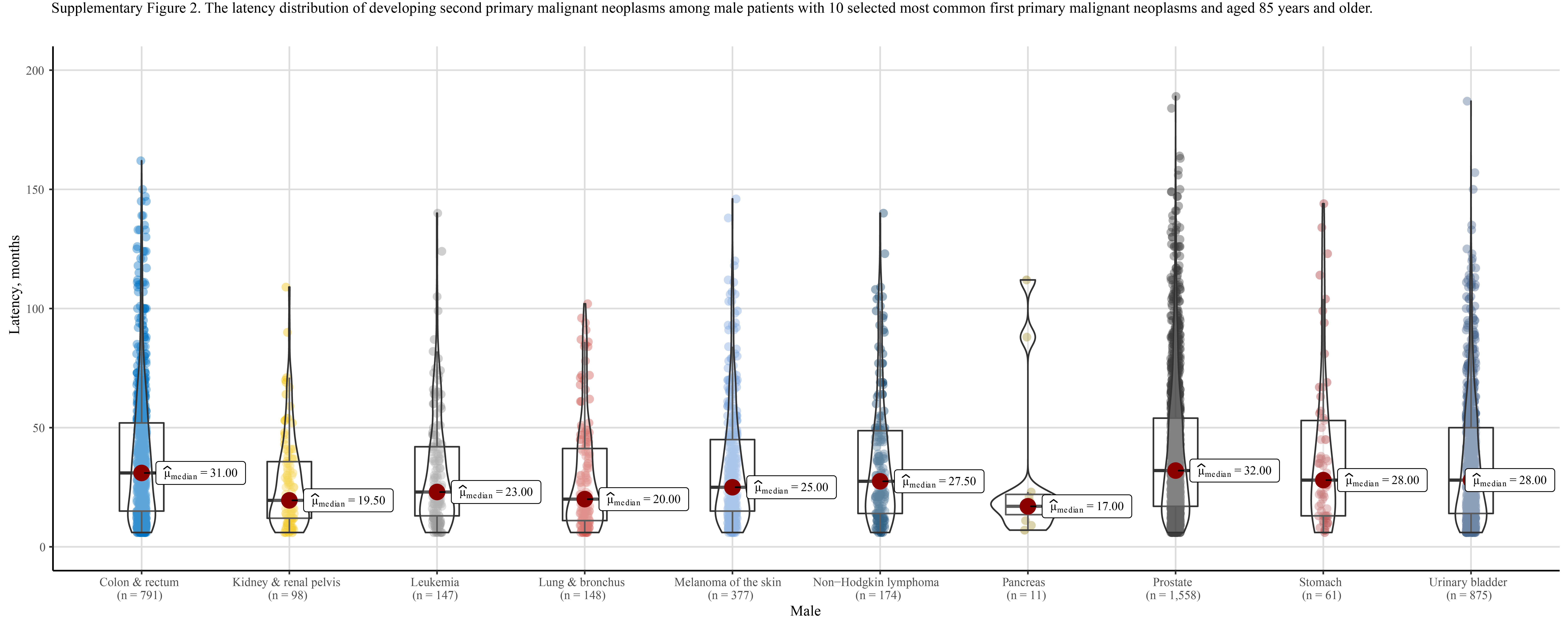

Supplement: Supplementary file 2 — Supplementary Information 2. [file 41598_2022_15746_MOESM2_ESM.jpg]

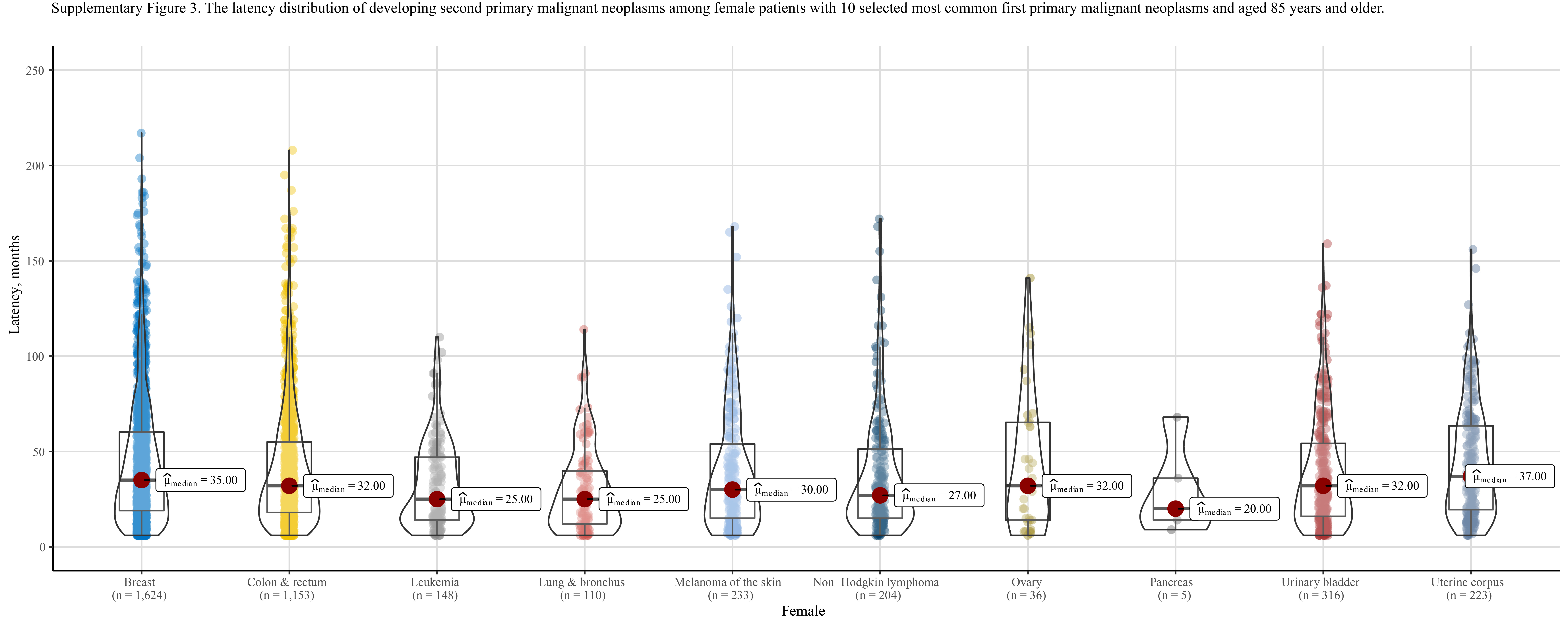

Supplement: Supplementary file 3 — Supplementary Information 3. [file 41598_2022_15746_MOESM3_ESM.jpg]
