# Supplementary material for: Epidemiological analysis of second primary malignant neoplasms in cancer survivors aged 85 years and older: a SEER data analysis (1975–2016)
Source: Sci Rep. 2022 Jul 8;12:11688. doi: 10.1038/s41598-022-15746-x (PMC9270446; doi:10.1038/s41598-022-15746-x)
Supplement: Supplementary file 4 — Supplementary Information 4. [file 41598_2022_15746_MOESM4_ESM.docx]

Supplementary Table 1: Baseline Clinical Features Comparison between OPM Group and SPM Group for Male Cancer Survivors, Ages 85 Years and Older, SEER, 1975-2016.

| **Variables** | **Total,**  **N=52336** | **OPM,**  **N=48096** | **SPM,**  **N=4240** | **P value** |
| --- | --- | --- | --- | --- |
| **Age, years** | 87 (86, 89) | 87 (86, 89) | 87 (85, 89) | *< 0.001* |
| **Race** |  |  |  | *< 0.001* |
| White | 46017 (88) | 42202 (88) | 3815 (90) |  |
| Black | 2592 (5) | 2409 (5) | 183 (4) |  |
| Others | 3727 (7) | 3485 (7) | 242 (6) |  |
| **FPM site** |  |  |  | *< 0.001* |
| Lung & bronchus | 3781 (7) | 3633 (8) | 148 (3) |  |
| Prostate | 21462 (41) | 19904 (41) | 1558 (37) |  |
| Urinary Bladder | 8113 (16 | 7238 (15) | 875 (21) |  |
| Colon & rectum | 9114 (17) | 8323 (17) | 791 (19) |  |
| Melanoma of the skin | 2935 (6) | 2558 (5) | 377 (9) |  |
| Non-Hodgkin lymphoma | 2372 (5) | 2198 (5) | 174 (4) |  |
| Leukemia | 1961 (4) | 1814 (4) | 147 (3) |  |
| Pancreas | 411 (1) | 400 (1) | 11 (0) |  |
| Kidney & renal pelvis | 806 (2) | 708 (1) | 98 (2) |  |
| Stomach | 1381 (3) | 1320 (3) | 61 (1) |  |
| **Grade** |  |  |  | *< 0.001* |
| I | 5114 (10) | 4674 (10) | 440 (10) |  |
| II | 15857 (30) | 14419 (30) | 1438 (34) |  |
| III | 13198 (25) | 12261 (25) | 937 (22) |  |
| IV | 2531 (5) | 2344 (5) | 187 (4) |  |
| Unknown | 15636 (30) | 14398 (30) | 1238 (29) |  |
| **SEER stage** |  |  |  | *< 0.001* |
| Local | 14215 (27) | 12551 (26) | 1664 (39) |  |
| Regional | 7030 (13) | 6502 (14) | 528 (12) |  |
| Distant | 5650 (11) | 5403 (11) | 247 (6) |  |
| Local/regional (Prostate cases only) | 8363 (16) | 7637 (16) | 726 (17) |  |
| Unknown | 17078 (33) | 16003 (33) | 1075 (25) |  |
| **Survival status** |  |  |  | *< 0.001* |
| Alive | 5588 (11) | 5265 (11) | 323 (8) |  |
| Died of cancer | 16668 (32) | 15835 (33) | 833 (20) |  |
| Died of non-cancer | 26789 (51) | 23945 (50) | 2844 (67) |  |
| Died of unknown reason | 3291 (6) | 3051 (6) | 240 (6) |  |
| Data are n (%), n/N (%), or median (IQR), unless specified otherwise.  OPM, one primary malignancy; SPM, second primary malignant neoplasms; FPM, first primary malignant neoplasms; SEER, Surveillance, Epidemiology, and End Results. | | | | |
