# Supplementary material for: Epidemiological analysis of second primary malignant neoplasms in cancer survivors aged 85 years and older: a SEER data analysis (1975–2016)
Source: Sci Rep. 2022 Jul 8;12:11688. doi: 10.1038/s41598-022-15746-x (PMC9270446; doi:10.1038/s41598-022-15746-x)
Supplement: Supplementary file 5 — Supplementary Information 5. [file 41598_2022_15746_MOESM5_ESM.docx]

Supplementary Table 2: Baseline Clinical Features Comparison between OPM Group and SPM Group for Female Cancer Survivors, Ages 85 Years and Older, SEER, 1975-2016.

| **Variables** | **Total,**  **N=67754** | **OPM,**  **N=63702** | **SPM,**  **N=4052** | **P value** |
| --- | --- | --- | --- | --- |
| **Age, years** | 88 (86, 90) | 88 (86, 90) | 87 (86, 89) | *< 0.001* |
| **Race** |  |  |  | *0.071* |
| White | 61143 (90) | 57492 (90) | 3651 (90) |  |
| Black | 3430 (5) | 3200 (5) | 230 (6) |  |
| Others | 3181 (5) | 3010 (5) | 171 (4) |  |
| **FPM site** |  |  |  | *< 0.001* |
| Breast | 25010 (37) | 23386 (37) | 1624 (40) |  |
| Colon & rectum | 19013 (28) | 17860 (28) | 1153 (28) |  |
| Lung & bronchus | 4034 (6) | 3924 (6) | 110 (3) |  |
| Pancreas | 853 (1) | 848 (1) | 5 (0) |  |
| Non-Hodgkin lymphoma | 3863 (6) | 3659 (6) | 204 (5) |  |
| Urinary bladder | 4453 (7) | 4137 (6) | 316 (8) |  |
| Leukemia | 2574 (4) | 2426 (4) | 148 (4) |  |
| Melanoma of the skin | 2807 (4) | 2574 (4) | 233 (6) |  |
| Corpus uteri | 3865 (6) | 3642 (6) | 223 (6) |  |
| Ovary | 1282 (2) | 1246 (2) | 36 (1) |  |
| **Grade** |  |  |  | *< 0.001* |
| I | 7656 (11) | 7071 (11) | 585 (14) |  |
| II | 21826 (32) | 20429 (32) | 1397 (34) |  |
| III | 12052 (18) | 11413 (18) | 639 (16) |  |
| IV | 2077 (3) | 1977 (3) | 100 (2) |  |
| Unknown | 24143 (36) | 22812 (36) | 1331 (33) |  |
| **SEER stage** |  |  |  | *< 0.001* |
| Local | 32766 (48) | 30282 (48) | 2484 (61) |  |
| Regional | 17713 (26) | 16765 (26) | 948 (23) |  |
| Distant | 8112 (12) | 7846 (12) | 266 (7) |  |
| Unknown | 9163 (14) | 8809 (14) | 354 (9) |  |
| **Survival status** |  |  |  | *< 0.001* |
| Alive | 8868 (13) | 8499 (13) | 369 (9) |  |
| Died of cancer | 20582 (30) | 19822 (31) | 760 (19) |  |
| Died of non-cancer | 32774 (48) | 30211 (47) | 2563 (63) |  |
| Died of unknown reason | 5530 (8) | 5170 (8) | 360 (9) |  |
| Data are n (%), n/N (%), or median (IQR), unless specified otherwise.  OPM, one primary malignancy; SPM, second primary malignant neoplasms; FPM, first primary malignant neoplasms; SEER, Surveillance, Epidemiology, and End Results. | | | | |
