# Supplementary material for: Epidemiological analysis of second primary malignant neoplasms in cancer survivors aged 85 years and older: a SEER data analysis (1975–2016)
Source: Sci Rep. 2022 Jul 8;12:11688. doi: 10.1038/s41598-022-15746-x (PMC9270446; doi:10.1038/s41598-022-15746-x)
Supplement: Supplementary file 6 — Supplementary Information 6. [file 41598_2022_15746_MOESM6_ESM.docx]

Supplementary Table 3. Leading Sites of SPM for Selected FPM, Ages 85 Years and Older, SEER, 1975-2016.

| **Male** | | | | | **Female** | | | |
| --- | --- | --- | --- | --- | --- | --- | --- | --- |
| **Site of FPM** | **Site of SPM** | | **N** | **%** | **Site of FPM** | **Site of SPM** | **N** | **%** |
| Lung & bronchus | Lung & bronchus | 28 | | 18.92% | Breast | Breast | 420 | 25.86% |
|  | Prostate | 27 | | 18.24% |  | Colon & rectum | 324 | 19.95% |
|  | Urinary bladder | 26 | | 17.57% |  | Lung & bronchus | 120 | 7.39% |
| Prostate | Colon & rectum | 367 | | 23.56% | Colon & rectum | Colon & rectum | 311 | 26.97% |
|  | Urinary bladder | 239 | | 15.34% |  | Breast | 229 | 18.28% |
|  | Lung & bronchus | 209 | | 13.41% |  | Lung & bronchus | 120 | 10.41% |
| Urinary bladder | Urinary bladder | 198 | | 22.63% | Lung & bronchus | Lung & bronchus | 28 | 25.45% |
|  | Prostate | 154 | | 17.60% |  | Colon & rectum | 17 | 15.45% |
|  | Lung & bronchus | 124 | | 14.17% |  | Breast | 15 | 13.63% |
| Colon & rectum | Prostate | 210 | | 26.55% | Pancreas | Colon & rectum | 3 | 60.00% |
|  | Colon & rectum | 128 | | 16.18% |  | Breast | 1 | 20.00% |
|  | Lung & bronchus | 93 | | 11.76% |  | Soft tissue | 1 | 20.00% |
| Melanoma of the skin | Melanoma of the skin | 128 | | 33.95% | Non-Hodgkin  lymphoma | Colon & rectum | 36 | 17.65% |
|  | Prostate | 69 | | 18.30% |  | Non-Hodgkin lymphoma | 28 | 13.73% |
|  | Lung & bronchus | 40 | | 10.61% |  | Breast | 25 | 12.25% |
| Non-Hodgkin lymphoma | Urinary bladder | 29 | | 16.67% | Urinary bladder | Urinary bladder | 61 | 19.30% |
|  | Colon & rectum | 25 | | 14.37% |  | Breast | 49 | 15.51% |
|  | Lung & bronchus | 19 | | 10.92% |  | Colon & rectum | 48 | 15.19% |
| Leukemia | Prostate | 39 | | 26.53% | Leukemia | Colon & rectum | 36 | 24.32% |
|  | Colon & rectum | 22 | | 14.97% |  | Breast | 22 | 14.86% |
|  | Lung & bronchus | 17 | | 11.56% |  | Lung & bronchus | 13 | 8.78% |
| Pancreas | Prostate | 3 | | 27.27% | Melanoma of  the skin | Melanoma of  the skin | 79 | 33.91% |
|  | Stomach | 3 | | 27.27% |  | Colon & rectum | 35 | 15.02% |
|  | Colon & rectum | 1 | | 9.09% |  | Breast | 34 | 14.59% |
| Kidney & renal pelvis | Urinary bladder | 43 | | 43.88% | Uterine corpus | Breast | 54 | 24.21% |
|  | Prostate | 13 | | 13.27% |  | Colon & rectum | 47 | 21.08% |
|  | Colon & rectum | 10 | | 10.20% |  | Lung & bronchus | 25 | 11.21% |
| Stomach | Prostate | 14 | | 22.95% | Ovary | Colon & rectum | 12 | 33.33% |
|  | Colon & rectum | 10 | | 16.39% |  | Breast | 8 | 22.22% |
|  | Urinary bladder | 9 | | 14.75% |  | Kidney & renal pelvis | 3 | 8.33% |
| SPM, second primary malignancy; FPM, first primary malignancy; SEER, Surveillance, Epidemiology, and End Results. | | | | | | | | |
