# Supplementary material for: Epidemiological analysis of second primary malignant neoplasms in cancer survivors aged 85 years and older: a SEER data analysis (1975–2016)
Source: Sci Rep. 2022 Jul 8;12:11688. doi: 10.1038/s41598-022-15746-x (PMC9270446; doi:10.1038/s41598-022-15746-x)
Supplement: Supplementary file 7 — Supplementary Information 7. [file 41598_2022_15746_MOESM7_ESM.docx]

Supplementary Table 4. Relative Post Second Primary Malignant Neoplasms Survival for Selected First Primary Malignant Neoplasms, Ages 85 Years and Older, SEER, 1975-2016.

| First primary  malignant neoplasms | 1-year | 3-year | 5-year |
| --- | --- | --- | --- |
| Female |  |  |  |
| Breast | 53% | 28% | 15% |
| Colon & rectum | 52% | 26% | 13% |
| Lung & bronchus | 47% | 20% | 8% |
| Pancreas | 60% | 20% | 20% |
| Non-Hodgkin lymphoma | 45% | 18% | 6% |
| Urinary bladder | 46% | 24% | 10% |
| Leukemia | 33% | 15% | 6% |
| Melanoma of the skin | 54% | 27% | 13% |
| Ovary | 46% | 23% | 9% |
| Uterine corpus | 43% | 24% | 13% |
| Male |  |  |  |
| Lung & bronchus | 42% | 16% | 8% |
| Prostate | 43% | 19% | 10% |
| Urinary bladder | 45% | 17% | 8% |
| Colon & rectum | 52% | 21% | 10% |
| Melanoma of the skin | 54% | 26% | 11% |
| Non-Hodgkin lymphoma | 42% | 18% | 6% |
| Leukemia | 33% | 12% | 7% |
| Pancreas | 27% | 9% | 9% |
| Kidney & renal pelvis | 56% | 27% | 16% |
| Stomach | 32% | 12% | 7% |

SEER, Surveillance, Epidemiology, and End Results
